# Novel Temporal Expression Patterns of EBF-Binding Proteins in Wing Morphs of The Grain Aphid *Sitobion miscanthi*

**DOI:** 10.3389/fphys.2021.732578

**Published:** 2021-08-26

**Authors:** Siyu Zhang, Qian Zhang, Xin Jiang, Qian Li, Yaoguo Qin, Wenkai Wang, Jia Fan, Julian Chen

**Affiliations:** ^1^State Key Laboratory for Biology of Plant Diseases and Insect Pests, Institute of Plant Protection, Chinese Academy of Agricultural Sciences, Beijing, China; ^2^School of Agriculture, Yangtze University, Jingzhou, China

**Keywords:** *Sitobion miscanthi*, odorant binding protein, expression pattern, temporal expression, E-β-farnesene (EBF), antenna

## Abstract

High chemosensitivity of insects to volatile organic compounds (VOC) stimuli is mediated by odorant binding proteins (OBPs). In aphids, three OBPs (OBP3, OBP7 and OBP9) are E-β-farnesene (EBF)-binding proteins. Winged aphids are generally more sensitive than wingless aphids to VOCs, thus, wing presence is a phenotypic correlate of olfaction sensitivity. Here, we investigate the detailed temporal expression of these EBF-binding proteins and two other *OBPs* (*OBP6* and *OBP10*), in the grain aphid *Sitobion miscanthi* 0 h, 2 h, 1 day, 3 days, 10 days, and 20 days after adult emergence. Both winged and wingless aphids were examined to further uncover phenotypic specification. Then, the expression patterns before and after EBF induction were analyzed. Throughout adulthood, only *OBP7* had significantly higher antennal expression in winged aphids; however, there was no significant difference in the antennal expression of *OBP3* between wing morphs at most time points. Except it was lower in newly emerged winged aphids but increased rapidly to the same level in wingless aphids at 1 day. *OBP9* did not differ in expression between the morphs and was the only OBP that did not exhibit an expression trough at the beginning of the adult stage (0 h). The expression of *OBP9* remained relatively stable and high throughout the adult stage in both phenotypes, showing the highest level among the three EBF-binding proteins. After EBF induction, its expression was further up-regulated in both morphs. Therefore, this protein may be an important molecule for EBF recognition in aphids. *OBP7* strongly responded to EBF but only in winged aphids, suggesting that this protein is important in the more sensitive EBF recognition process of winged aphids. In addition, the antennal expression level of *OBP3* did not respond to EBF induction. These findings revealed a temporal expression pattern of *OBPs* in aphids and showed that figuring out the pattern is critical for correctly selecting morphs and sampling times, which will support the discovery of reliable findings and allow solid conclusions to be drawn. Our findings also inspire on the interaction mode of the three EBF-binding proteins in relation to EBF perception in aphids.

## Introduction

Odorant binding proteins (OBPs) with high antennal expression levels have been widely identified among insect species since 1981 (Vogt and Riddiford, 1981). The widespread distribution of these proteins, together with their broad affinities for plant volatiles as well as pheromones *in vivo*, suggest critical roles in peripheral signal transmission for foreign olfactory ligands. Compared with other insects, aphids generally express fewer OBPs (between nine and twelve), such as *Acyrthosiphon pisum* with 11 identified genes encoding complete OBPs (Zhou et al., 2010; Biasio et al., 2015), *Aphis gossypii* with nine (Gu et al., 2013), *Myzus persicae* with 11 (Ji et al., 2016), *Sitobion avenae* with twelve (Xue et al., 2016), *Megoura viciae* with 10 (Daniele et al., 2018), and *Aphis glycines* with 11 (Wang et al., 2021). However, at least three OBPs, namely, OBP3 (Qiao et al., 2009), OBP7 (Sun et al., 2012a) and OBP9 (Qin et al., 2020), exhibit properties as E-β-farnesene (EBF)-binding proteins in aphids. The release of alarm pheromone signals enables aphids to escape and defend themselves (Nault et al., 1973; Pickett and Griffiths, 1980). The high proportion of EBF-binding proteins (three out of the nine to twelve OBPs) indicates the importance of alarm pheromones in aphid survival and population expansion.

Spatial expression profiles showed that insect *OBPs* tended to be expressed in chemosensory organs such as antennae, heads and legs (e.g., Vogt and Riddiford, 1981; Sun et al., 2013). Besides, they occur in non-sensory tissues and organs such as the wings (Calvello et al., 2003; Pelosi et al., 2005), reproductive organs (Li et al., 2008; Sun et al., 2012a), mandibular glands (Iovinella et al., 2011) and salivary glands (Zhang et al., 2017) as well. Work on OBPs in aphids has also been widely published, including studies focusing on expression analysis between winged and wingless morphs (e.g., Gu et al., 2013; Xue et al., 2016; Wang et al., 2019, 2021). For example, Wang et al. (2021) conducted a systematic and detailed investigation of *OBP* expression in the antennae, head, wings, legs, cornicles, caudae, and thorax of the soybean aphid *A. glycines* through differential transcriptome analysis and quantitative real-time PCR (RT-qPCR).

The temporal expression profile of aphids has mainly been compared between the nymph and adult stages (e.g., Xue et al., 2016; Wang et al., 2019). The expression profiles revealed higher levels of some *OBPs* in the nymph stage than in adults. However, considering that the body undergoes a substantial transformation from the nymph to adult stage and that the adult stage lasts up to an average of 19 d (Li et al., 2018), a detailed investigation of key time points such as the beginning of the adult stage and several time points during the whole adult stage will hopefully clarify the temporal expression patterns of *OBPs* throughout the adult stage. To date, there has been a lack of research on this topic.

Wing state is a phenotypic correlate of olfaction sensitivity in aphids, with the winged morph displaying greater sensitivity (Pickett, 2009). Winged aphids play more important roles in risk avoidance, migration, and habitat reselection over long distances, whereas wingless aphids are responsible for rapid population expansion after colonization. Therefore, additional investigations of the expression pattern of *OBPs* between the two wing phenotypes will refine the study of temporal expression patterns. In addition, such investigations would help reveal the potential function of OBPs from a novel perspective.

Insect hyperawareness of potential food, mates or danger is mostly due to the highly sensitive insect chemosensory system. Intense responses of OBPs to chemical stimuli have been widely reported (e.g., Pelosi et al., 2005; Siciliano et al., 2014), but how intense the response of OBPs to newly occurring odors is in aphids remains an open question. Before answering this question, we must answer a more fundamental one: under constant conditions, what are the temporal expression patterns of *OBPs* during the aphid adult stage?

In the present study, we carried out a detailed investigation of the temporal expression profile of EBF-binding proteins in the grain aphid *Sitobion miscanthi*, the most widespread and harmful pest and dominant aphid species of wheat in China, at the beginning, middle and end of the adult stage. In China, *S. miscanthi* was wrong using for the Latin name as *S. avenae* (Zhang, 1999; Jiang et al., 2019). The time points employed in the survey were 0, 2 h, 1 day, 3 days, 10 days, and 20 days after the emergence of adults. Furthermore, we collected antennae from both winged and wingless individuals to determine whether wing morph is a phenotypic correlate of *OBP* expression. Finally, the expression of these genes before and after EBF induction was analyzed by carrying out an EBF treatment experiment.

## Materials and Methods

### Aphid Samples

The grain aphid *S. miscanthi* clone was originally collected from wheat in Hebei Province, China, and kept in our laboratory, which is not privately owned or protected. An isogenic colony was started from a single parthenogenetic female and was maintained on wheat (*Triticum aestivum*) in the laboratory at 22 ± 1°C with a 75% relative humidity and 16 h light/8 h dark. Our recent investigation showed an average adult longevity of 19 d in *S. miscanthi* (Li et al., 2018), which indicates that 20 d after adult emergence would be the very end of life.

### Sampling

In the present study, we chose 0 h, 2 h (2 h ± 5 min), 1 day (24–36 h), and 3 days (72–84 h) after adult emergence as the beginning of the adult stage, 10 d as the middle of adult stage and 20 d as the end of adult stage to explore the temporal expression patterns of *OBPs* in *S. miscanthi*.

Fourth-instar (the last nymph stage) winged and wingless nymphs were placed in 9-cm-diameter Petri dishes (10 aphids/dish) and fed wheat seedlings grown in 2 ml centrifuge tubes. Antennae at each time points were collected from both wingless and winged aphids. For the EBF treatment experiment, 4th-instar winged and wingless nymphs were placed into 9-cm-diameter Petri dishes (10 aphids/dish) lined with filter paper moistened with water. The nymphs were fed wheat seedlings grown in 2 ml centrifuge tubes. Twenty-four hours after they emerged as adults, 400 ng of EBF (1 μL 400 ng/μL, diluted with *n*-hexane) was applied to a 1 cm square filter paper and quickly placed into the Petri dish. Filter paper (1 cm) with 1 μl of n-hexane served as a control. Sampling was performed after 30 min of treatment. Thirty-five pairs of antennae were dissected into 1.5 ml centrifuge tubes in triplicate for each time point as well as EBF treatment and immediately frozen in liquid nitrogen. Antenna samples were ultimately stored at −80°C before total RNA extraction.

### Reagents

E-β-farnesene was purchased from Wako Chemical (Osaka, Japan), and n-hexane was purchased from Sigma-Aldrich (St. Louis, MO, United States).

### Total RNA Extraction

Total RNA was extracted using total RNA extraction reagent (Tianmo, Beijing, China) following the manufacturer’s instructions. The concentration of RNA (OD260/OD280 value) was measured by a Nanodrop 2000 spectrophotometer (DeNovix, Washington, DC, United States). Then, the first strand of cDNA was synthesized using 400 ng of total RNA using the 1st strand cDNA synthesis kit (aidlab, Beijing, China).

### Quantitative Real-Time PCR Analysis

The antennal expression levels of *OBP3, OBP6, OBP7, OBP9*, and *OBP10* transcripts at different time points of the adult stage as well as in the EBF treatment experiment were quantified by RT-qPCR. The sequences of primers used for RT-qPCR were designed based on *SaveOBP3* (GenBank accession number KU140607), *SaveOBP6* (KU140610), *SaveOBP7* (KU140611), *SaveOBP9* (KU140613) and *SaveOBP10* (KU140614), which were previously reported by Xue et al. (2016) and are listed in Supplementary Table 1. The system was established according to the instructions of the SuperReal PreMix Plus Kit (SYBR Green) (Tiangen, Beijing, China). The qPCR reactions were prepared at a total volume of 20 μl with 2 μl of cDNA and 0.5 μl of each primer. qPCR was performed on an ABI 7500 Real-Time PCR System (Applied Biosystems, Carlsbad, CA, United States). The parameters for qPCR amplification were 95°C for 30 s, followed by 40 cycles of 95°C for 15 s and 60°C for 30 s.

To normalize target gene expression and correct for sample-to-sample variation, the relative quantities were calculated based on two internal control genes (Vandesompele et al., 2002), NADH dehydrogenase and dimethyladenosine transferase (DIMT), which were identified from antennal transcriptome data of *S. avenae* (Xue et al., 2016).

The five target genes and two internal control genes were amplified in each sample under the same conditions, and each gene was analyzed using three technical replicates and three biological replicates.

The expression levels of *OBP3, OBP6, OBP7, OBP9* and *OBP10* in the antennae of both winged and wingless aphids at different time points after adult emergence were calculated and statistically analyzed. The temporal expression pattern of each *OBP* among the five time points was analyzed using the value from the antennae of wingless aphids at 0 h as the external reference. The expression of the five *OBPs* at a given time point was relative to the transcript level of *OBP9* in the antennae of wingless aphids at the corresponding time point.

The expression levels of O*BP3, OBP6, OBP7, OBP9*, and *OBP10* in the antennae of winged and wingless aphids before and after EBF induction were quantified relative to the transcript levels of *OBP9* in winged aphids and *OBP9* in wingless aphids, respectively.

### Statistical Analysis

Differences in transcript expression at different time points of adult aphid emergence were tested by one-way analysis of variance (ANOVA) followed by Duncan’s multiple range test using SPSS version 23.0 software (IBM, Armonk, NY, United States). Differences in antennal transcript expression between winged and wingless aphids were analyzed by two-sample *t*-tests.

## Results

### Expression Patterns

**FIGURE 1:**
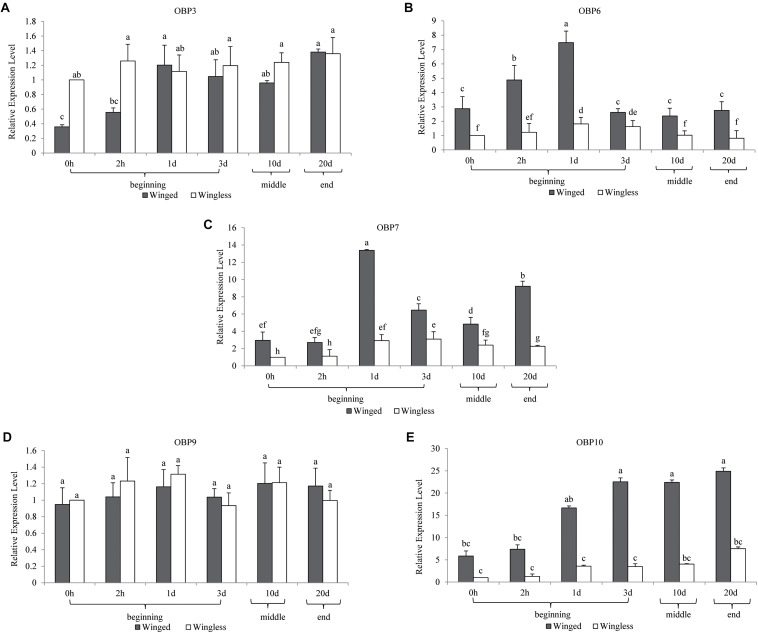
Temporal expression profiles of *OBP3/6/7/9/10* in *S. miscanthi*. Fold changes are relative to the antennal transcript levels of the wingless morph at 0 h after adult emergence. Differences in mean transcript levels were detected using one-way ANOVA, followed by Duncan’s multiple range test. Different letters over bars indicate significant differences (*p* < 0.05). Winged, antennae of winged aphid; Wingless, antennae of wingless aphid; Beginning, middle, end, the adult stage after emergence was divided into three parts, namely, beginning (within 3 d), middle (10 d), and end (20 d).

**FIGURE 2:**
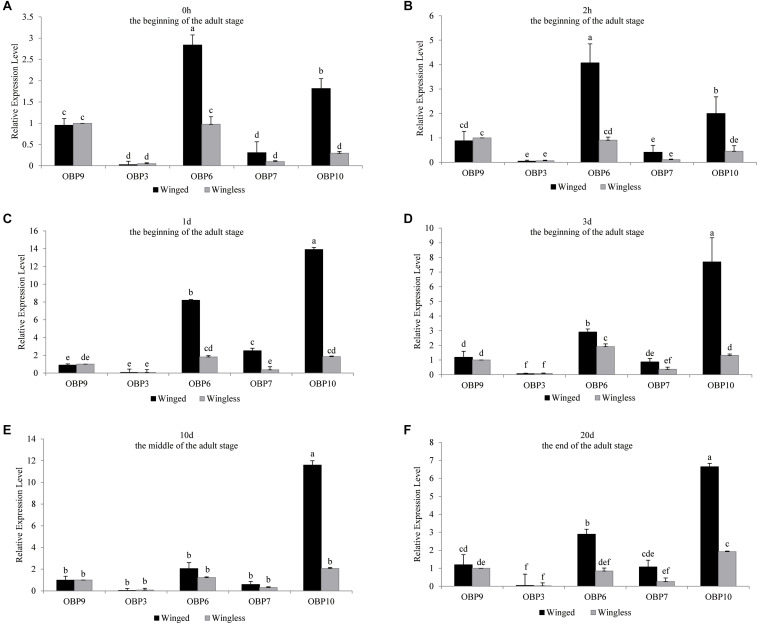
*OBP3/6/7/9/10* expression at each time point (0 h, 1 d, 3 d, 10 d, and 20 d) in *S. miscanthi*. Fold changes are relative to the antennal transcript levels of OBP9 in the wingless morph at the corresponding time point. Differences in mean transcript levels were detected using one-way ANOVA, followed by Duncan’s multiple range test. Different letters over bars indicate significant differences (*p* < 0.05). Winged, antennae of winged aphid; Wingless, antennae of wingless aphid; Beginning, middle, end, the adult stage after emergence was divided into three parts, namely, beginning (within 3 d), middle (10 d), and end (20 d).

There was no significant difference in the antennal expression of *OBP3* between winged and wingless aphids at most time points (Figure 1). However, at the beginning of the adult stage (0 h), *OBP3* expression in the antennae of the winged type was significantly lower than that in the wingless type, after which it increased to the level observed in the wingless type at 1 day and remained stable in the later adult stage. Meanwhile, the antennal expression of *OBP3* in wingless aphids remained stable after adult emergence (at the *P* = 0.05 level, Figure 1). Furthermore, as shown in Figure 2, the expression level of *OBP3* in the antennae of both morphs was much lower than that of the other four *OBPs* (*OBP6/7/9/10*, at the *P* = 0.05 level, Figure 2).

The antennal expression of *OBP6* in the winged morph was higher than in the wingless morph at all detection time points. However, the expression in both morphs showed a consistent trend over time. It peaked at the same time point (1 d) in the two morphs and then decreased to the level observed at 0 h (at the *P* = 0.05 level, Figure 1).

Although the antennal expression of *OBP7* in the wingless morph increased at 1 day and then remained significantly higher than that at 0 h in the later adult stage (at 1 day, 3 days, 10 days, and 20 days, Figure 1), it was lower than that in the winged morph at all time points. The antennal expression of *OBP7* in the winged morph increased rapidly after 2 h and reached a peak at 1 day. After that, although it fluctuated, it stayed higher than the expression at 0 h as well as the expression in the wingless morph, as mentioned above (at the *P* = 0.05 level, Figure 1).

The expression level of *OBP9* remained stable throughout the whole adult stage, including at the beginning of emergence, and there was no significant difference between the winged and wingless phenotypes (at the *P* = 0.05 level, Figure 1).

The antennal expression level of *OBP10* in the wingless morph, which was far lower than that in the winged morph in adulthood (Figure 1), showed an upward trend, but there was no statistically significant change during the adult stage. The level in winged aphids was lower at the beginning of adult emergence; it increased rapidly and remained stably high after 2 h and throughout the later adult stage (at the *P* = 0.05 level, Figure 1). Moreover, our analysis results showed that among the five tested *OBPs*, *OBP10* showed the highest antennal expression in both winged and wingless antennae after time point “2 h” (at the *P* = 0.05 level, Figure 2).

According to the above results, among the three EBF-binding proteins, *OBP3* and *OBP9* were stable and not differentially expressed in the two phenotypes. *OBP7* was highly expressed in winged aphids, showing significant phenotypic specificity. It is also worth noting that in contrast to other *OBPs*, *OBP3* was significantly more highly expressed in the wingless morph at 0 h.

### EBF Induction

**FIGURE 3:**
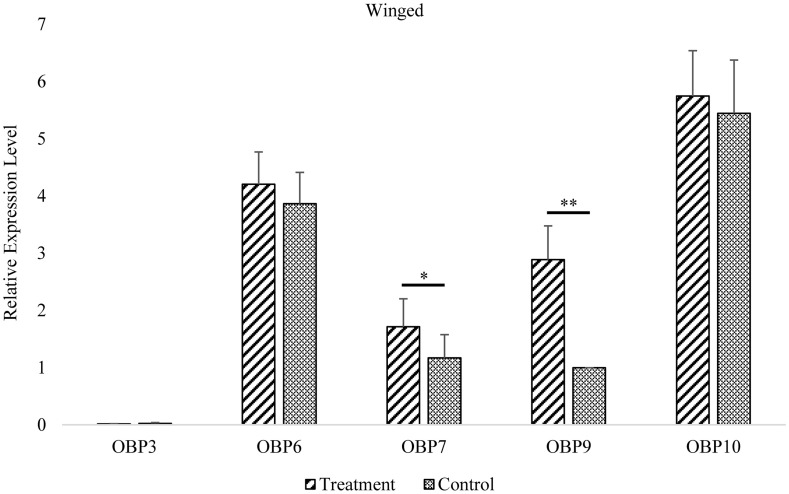
Antennal *OBP3/6/7/9/10* expression in the winged morph before and after EBF induction. Fold changes are relative to the antennal transcript levels of OBP9 in the winged morph before EBF induction. *Significant difference at the *P* = 0.05 level. **Significant difference at the *P* = 0.01 level (two-sample *t*-test). Winged, antennae of winged aphid; Treatment, EBF induction; Control, n-hexane control.

**FIGURE 4:**
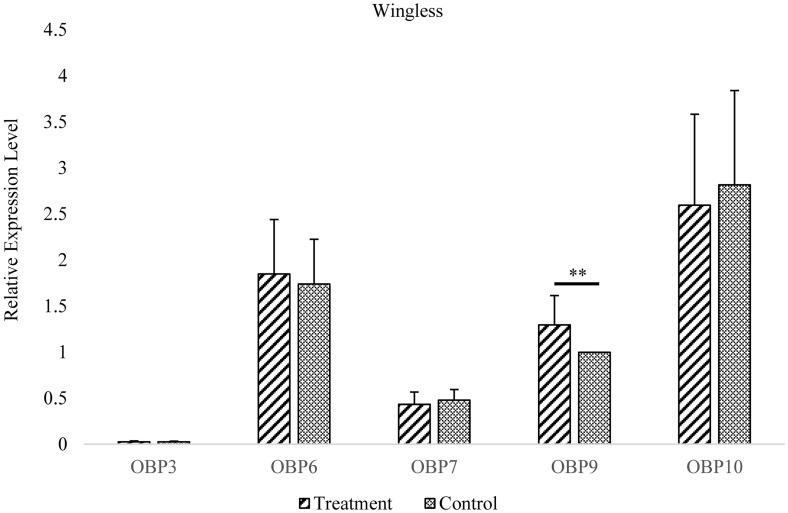
Antennal *OBP3/6/7/9/10* expression in the wingless morph before and after EBF induction. Fold changes are relative to the antennal transcript levels of OBP9 in the wingless morph before EBF induction. **Significant difference at the *P* = 0.01 level (two-sample *t*-test). Wingless, antennae of wingless aphid; Treatment, EBF induction; Control, blank control.

In wingless aphids, only *OBP9* expression was upregulated after induction by EBF (*P* < 0.01, Figure 3 and Supplementary Table 2). In winged aphids, *OBP9* expression was upregulated by EBF as well (*P* < 0.01, Figure 4 and Supplementary Table 2). In addition, the expression of another EBF-binding protein, *OBP7*, was found to be upregulated by EBF induction (*P* < 0.05, Figures 3, 4 and Supplementary Table 2) as well. Surprisingly, at the expression level, *OBP3*, one of the reported EBF-binding proteins, did not show any change after EBF induction in either wing morph (*P* > 0.05, Figures 3, 4 and Supplementary Table 2). *OBP6* and *OBP10* showed patterns similar to that of *OBP3*, and there was no significant difference between the EBF treatment and control in either morph (*P* > 0.05, Figures 3, 4 and Supplementary Table 2).

## Discussion

OBP3 in the pea aphid *A. pisum* was the first reported EBF-binding protein as well as the first OBP reported in aphids (Qiao et al., 2009). Several studies have since reported that OBP7 also shows a specific affinity for EBF in the pea aphid *A. pisum* and peach aphid *M. persicae* (Sun et al., 2012b), grain aphid *S. avenae* (Zhong et al., 2012) and bird cherry oat aphid *Rhopalosiphum padi* (Fan et al., 2017). More recently, OBP9 was reported to have a broad affinity spectrum including EBF (Qin et al., 2020). To date, at least three EBF-binding proteins have been reported, which suggests that peripheral transmission of EBF may be achieved through the interaction of multiple OBPs.

In the present study, in early adulthood, we found two trends of *OBP* expression, namely, stable expression (*OBP3* in the wingless morph, *OBP9* in the winged and wingless morphs, and *OBP10* in the wingless morph, Figure 1) and upregulated expression (*OBP3* and *OBP10* in the winged morph and *OBP6* and *OBP7* in the winged and wingless morphs, Figure 1), and there was no downregulated expression. By late aphid adulthood, even including the very end of the life cycle (20 d), the expression levels of the five *OBPs* all remained steady. Our investigation revealed complex expression level patterns for different *OBPs* during the aphid adult stage. Therefore, to clarify the expression patterns of aphid *OBPs*, we suggest subdividing the adult stage into different periods and including 0 h as a sampling time point. An additional odorant, i.e., EBF, was able to further stimulate the expression of corresponding *OBPs*, which obviously increased the complexity of the expression patterns.

Interestingly, among the three EBF-binding proteins, only *OBP7* was significantly more highly expressed in the antennae of winged aphids than in wingless aphids. Within the investigated temporal range, the antennal expression levels of this protein were regularly dynamic but remained higher in the winged morph than in the wingless morph at all time points. However, there was a lower but continuously stable expression level in the wingless morph. However, *OBP3* was found to have a novel expression pattern between the wing morphs. There was a significantly lower level of antennal *OBP3* expression in newly emerged winged aphids (at the *P* = 0.05 level), which increased rapidly to the same level as that in the wingless aphids at 1 day and remained steady and equivalent to that level over time. This is in sharp contrast with the patterns observed for *OBP6*, *OBP7*, and *OBP10* (sustained higher expression level in the winged morph, Figure 1) and *OBP9* (sustained high expression level in both wing morphs with no significant difference, Figure 1). Similar to that of *OBP3*, the expression of *OBP9* showed no phenotypic difference. *OBP9* was the only *OBP* that did not exhibit an expression trough at the beginning of the adult stage (0 h). This *OBP* showed relatively stable and high expression throughout the adult stage and the highest expression level among the three EBF-binding proteins (Figure 2). After EBF induction, the expression of this *OBP* was further upregulated in both morphs (Figures 3, 4). The above results indicated that this protein may be an important molecule for EBF recognition in aphids among two wing morphs. Antennal expression of *OBP7* strongly responded to EBF but only in the winged morph (Figures 3, 4), suggesting that this protein plays an important role in the more sensitive EBF recognition process of winged aphids. In addition, the expression level of *OBP3* in antennae did not respond to EBF induction. It remained at a low level in the antennae of both the winged and wingless phenotypes (Figures 1–4). According to previous work showing that *OBP3* is a systemic *OBP* with stronger expression in multiple tissues and organs, such as the cornicles and caudae (Xue et al., 2016; Wang et al., 2021), we speculated that *OBP3* may play a limited role in the olfactory recognition of EBF and may play a carrier role for EBF in EBF storage organs, such as cornicles. The phenotypic specificity of *OBP* expression has been reported in different studies with various results, showing highly complex patterns that are difficult to parse. For example, the expression level of *OBP7* in the winged morph did not increase to a relatively high level until 1 day. Assuming the sampling time is 0 or 2 h or a mixture of samples is sampled at various time points, the results may vary or even seem contradictory. Furthermore, the antennal expression level of *OBP7* in the winged aphids at 0 or 2 h (before the peak at 1 day) was the same as that in the wingless aphids during the whole adult stage after 1 day. Thus, once samples at these two time points were selected, it was not surprising that *OBP7* expression was not significantly different between winged and wingless aphids, as previously reported in some studies (e.g., Xue et al., 2016; Wang et al., 2019). As after emergence, most OBP genes undergo a process of increased expression and then stabilized, and the time to stabilize was about 1–3 days after emergence (Figure 1). Our analysis lead to the conclusion that treating the whole adult stage as a single stage in studies on expression levels does not provide sufficient resolution. In general, the repeatable and reliable expression level can be obtained 1–3 days after adult emergence when the OBP expression level is stable.

Similar to that of *OBP7*, the expression of *OBP6* peaked at 1 day in both morphs and then remained steady in the later adult stage, and *OBP10* presented expression patterns similar to that of *OBP7* as well (Figures 1, 2).

In summary, our findings will be helpful for understanding the interaction mode of the three EBF-binding proteins mediating EBF perception in aphids. Obviously, both phenotypes possess a molecular basis (*OBP9*) for ordinary EBF perception by the antennae. Furthermore, *OBP7* may enable greater sensitivity to EBF in winged aphids because of its significantly higher expression level in these aphids than in wingless aphids. Further functional studies are needed to clarify why *OBP3*, which has an EBF affinity, does not respond to EBF induction.

The above findings revealed the detailed temporal expression patterns of *OBPs* in aphids. They showed that figuring out the temporal expression patterns is critical for correctly selecting morphs and sampling times and will help researchers obtain reliable findings and draw solid conclusions.

## Data Availability Statement

The original contributions presented in the study are included in the article/Supplementary Material, further inquiries can be directed to the corresponding author/s.

## Author Contributions

JF conceived and designed the study. JF and JC supervised the project. SZ and QZ carried out the laboratory work. SZ, JF, XJ, YQ, and WW performed the RT-qPCR data analysis. SZ, JF, XJ, and QL worked on the statistical analysis and charting. JF and SZ wrote the original manuscript. All authors contributed on polishing and revising the work.

## Conflict of Interest

The authors declare that the research was conducted in the absence of any commercial or financial relationships that could be construed as a potential conflict of interest.

## Publisher’s Note

All claims expressed in this article are solely those of the authors and do not necessarily represent those of their affiliated organizations, or those of the publisher, the editors and the reviewers. Any product that may be evaluated in this article, or claim that may be made by its manufacturer, is not guaranteed or endorsed by the publisher.
