# Supplementary material for: Novel Temporal Expression Patterns of EBF-Binding Proteins in Wing Morphs of The Grain Aphid Sitobion miscanthi
Source: Front Physiol. 2021 Aug 26;12:732578. doi: 10.3389/fphys.2021.732578 (PMC8427609; doi:10.3389/fphys.2021.732578)
Supplement: Supplementary file 1 [file Table_1.DOCX]

**Table S1** Sequences of primers used for real‐time PCR

| **Primer name** | **Sense (5′ to 3′)** | **Antisense (3′ to 5′)** |
| --- | --- | --- |
| **SmisOBP3** | ACCATCGACAGAAACCGGAA | CGTAGACCATTCCGACCAGT |
| **SmisOBP6** | TTGCGATCATCTGCCAAACA | CCGGGCTTGGAATGAGAGTT |
| **SmisOBP7** | GAGTGAAGCGGCCATTAAAA | TGCCATCATCGTCATCTTGT |
| **SmisOBP9** | ACCTGCGAAGTTCCTCGAAT | GTTCTTTCAGTGCTGGCGAT |
| **SmisOBP10** | AAAGACACTGTACAATGCGTG | GGATGACTTGCTCGAAGATC |
| **NADH dehydrogenase** | CGAGGAGAACATGCTCTTAGAC | GATAGCTTGGGCTGGACATATAG |
| **Dimethyladenosine transferase (DIMT)** | GCCGTTTATCAATCAACAC | GGTGGATTACGAGGTTCTAT |
