# Supplementary material for: Novel Temporal Expression Patterns of EBF-Binding Proteins in Wing Morphs of The Grain Aphid Sitobion miscanthi
Source: Front Physiol. 2021 Aug 26;12:732578. doi: 10.3389/fphys.2021.732578 (PMC8427609; doi:10.3389/fphys.2021.732578)
Supplement: Supplementary file 2 [file Table_2.DOCX]

**Table S2** Detailed statistical results for the comparison between the EBF treatment and control

| *OBPs* | | *OBP3* | *OBP6* | *OBP7* | *OBP9* | *OBP10* |
| --- | --- | --- | --- | --- | --- | --- |
| *t* test (T and C) | Winged | ns, *P =* 0.123 | ns, *P* = 0.578 | *, *P* = 0.012 | **, *P* = 0.005 | ns, *P* = 0.296 |
|  | Wingless | ns, *P* = 0.620 | ns, *P* = 0.196 | ns, *P* = 0.563 | **, *P* = 0.007 | ns, *P* = 0.230 |

“T”: EBF treatment. “C”: control. “ns”: not significant. “*”: significant difference at the *P*=0.05 level. “**”: significant difference at the *P*=0.01 level. The expression levels in the treatment and its control were compared by a two-sample *t* test.
